# Tangential Ultrasonic-Vibration Assisted Forming Grinding Gear: An Experimental Study

**DOI:** 10.3390/mi13111826

**Published:** 2022-10-26

**Authors:** Wenbo Bie, Bo Zhao, Guofu Gao, Fan Chen, Jiangwei Jin

**Affiliations:** 1 School of Electrical and Mechanical Engineering, Pingdingshan University, Pingdingshan 467000, China; 2 School of Mechanical and Power Engineering, Henan Polytechnic University, Jiaozuo 454003, China; 3 Zhejiang Xinrui Welding Technology Co., Ltd., Shaoxing 312400, China

**Keywords:** gear, ultrasonic vibration-assisted grinding, grinding force, grinding temperature, residual stress, micromorphology

## Abstract

This study used a forming grinding wheel to machine an involute spur gear with ultrasonic vibration applied to the gear in order to improve the gear processing technology and enhance the gear processing effect. Conventional grinding and ultrasonic vibration-assisted forming grinding gear (TUVA-FGG) tests were carried out. The effects of grinding parameters, such as spindle speed, feed rate, radial grinding depth, and ultrasonic amplitude, on grinding force, grinding temperature, residual stress, surface roughness, and surface morphology were analyzed. The TUVA-FGG significantly improved processing efficiency. With the increase in spindle speed, the maximum reductions in the normal and tangential grinding forces, grinding temperature, and surface roughness reached 33.6, 24.5, 23.9, and 21.6%, respectively. With the increase in feed rate, the respective maximum reductions were 21.4, 19.7, 20.3, and 16.1%. With the increase in radial grinding depth, these values attained 24.6, 20.3, 21.5, and 17.6%, respectively. Finally, with the increase in ultrasonic amplitude, these reductions reached 21.4, 19.7, 21.5, and 19.4%. The maximum residual compressive stress grew by 30.3, 27.5, 30.9, and 27.5% with the increase of wheel rotation speed, feed speed, radial grinding depth, and ultrasonic amplitude, respectively. TUVA-FGG changed the conventional continuous cutting mechanism between the abrasive grain and workpiece into intermittent cutting, reducing grinding forces, grinding temperature, and surface roughness. Moreover, it increased residual compressive stress and improved the micromorphology of the tooth surface, thus enhancing gear machining efficiency.

## 1. Introduction

Gears are key components of movement- and power-transferring complex systems. Under service conditions, gears bear tensile and compressive alternating loads, as well as shear and dynamic stresses, leading to gear root bending fatigue, tooth surface contact fatigue, and wear and corrosion failure [1,2]. With the development of science and technology, the demand for gears in high-speed transport, aerospace, new energy, and other high-end equipment is increasing, putting forward higher and harsh requirements for gear manufacturing. In practical applications, once gear failure occurs, the dynamic performance of the gear transmission system will be slightly affected but may trigger gear tooth breakage, transmission system damage, power transmission interruption, and even machine destruction with serious safety problems and catastrophic accidents [3,4,5]. Therefore, manufacturing high-performance and long-life gears is crucial for equipment innovation and manufacturing level upgrades for high-end equipment in complex working conditions.

Ultrasonic-assisted machining, as an effective processing method applicable to various materials, can reduce the cutting forces, prolong the tool’s service life, enhance the machining accuracy, and improve surface topography, wear resistance, and corrosion resistance [6,7,8,9]. For the description of ultrasonic-assisted machining gear, in the 1970s, Kumabe [10] introduced the processing of ultrasonic vibration cutting, shaving, hobbing, grinding, and rolling gears. Subsequently, the application of ultrasonic machining to gear processing has been extended by numerous scholars who explored ultrasonic hobbing gear, lapping gear, honing gear, ultrasonic electrochemical gear processing, and ultrasonic hardening gear. Yin and Zhang [11] carried out ultrasonic vibration-assisted hobbing and conventional hobbing gear tests using a YM3150 precision hobbing machine. It was found that under the action of ultrasonic vibration, the cutting force and cutting temperature were significantly reduced, the build-up edges were restrained, and the gear surface roughness was decreased by one to two grades. Agapov [12,13] used an ultrasonic vibration device as an accessory to carry out ultrasonic hobbing gear tests, linking the cutting speed and machining efficiency, obtaining a surface roughness of *R_a_* = 0.68 μm, and improving the tool life by 1.6–1.8 times compared to conventional hobbing. Venkatesh [14] adopted ultrasonic-assisted abrasive flow machining to process the bevel gear and reported that the extension of processing time obviously improved the gear tooth surface quality, micromorphology, and texture smoothness. Wei et al. [15,16] developed a patent for gear lapping machining and theoretically analyzed the material removal mechanism and the impact and cavitation effects of cutting fluid on the tooth surface quality. Lü and Wang et al. [17,18] proposed a bending vibration-assisted honing gear, and the abrasive grain’s cutting speed was greatly enhanced under the action of high-frequency vibration, resulting in a significant reduction in the honing force. In addition, the accompanying cavitation effect of the cutting fluid improved the clogging of the honing wheel, reduced the tooth surface roughness, and improved the accuracy of the tooth. Pa [19] used ultrasonic-aid electrochemical finishing to machine gears and found that the gear surface can be smooth and bright, and the processing time can be shorter than that of conventional polishing. Jia et al. [20] investigated ultrasonic combined synchronous pulse electrochemical machining for microgears, with tooth profile accuracy up to ±0.005 mm and tooth surface roughness *R_a_* up to 0.16 μm. Singh [21,22] adopted ultrasonic electrochemical honing (UECH) to machine a bevel gear. He found that the average surface roughness and maximum surface roughness increased with the ultrasonic frequency and reached their optimal values of 0.31 and 6.88 mm, respectively, at an ultrasonic frequency of 42 kHz. Hattori et al. [23] carried out ultrasonic shot peening (USP) treatment on the gear tooth surface and tooth root, and the results showed that it could effectively improve surface finish and surface quality, reduce or avoid later polishing, and increase the residual compressive stress on the tooth surface 2–4 times, thus improving the anti-fatigue ability of the gear. Lan [24] employed the ultrasonic vibration-assisted rolling super gear root and found that its bending fatigue strength increased two times, and the bending fatigue life increased 15 times compared with conventional rolling. Jiang [25] investigated the longitudinal-torsional ultrasonic rolling gear tooth surface, and it was proven to be an effective method to improve gear contact fatigue, which was increased by 58% compared with that without rolling. The above studies show that the surface characteristics and fatigue resistance of gears are improved significantly under the action of ultrasonic vibration. Ultrasonic vibration-assisted grinding, as an effective means to control the energy input in the manufacturing process, can achieve high-performance manufacturing by precisely controlling the input interface energy. However, few studies have been conducted on the influence of ultrasonic vibration-assisted grinding on gear machining. It was attributed that the grinding parameters along the tooth profile are constantly changing, which leads to the complex geometry and physical multifield coupling state between the grinding wheel and tooth surface. To explore the effect of ultrasonic vibration-assisted grinding on the gear, in a previous study, ultrasonic vibration was applied on the gear workpiece and adopted the form of a grinding wheel machine spur gear. Compared with conventional grinding, ultrasonic vibration can reduce the surface roughness and improve the compressive residual compressive stresses to a certain extent [26,27]. However, the influence of processing parameters on the grinding force, grinding temperature, and surface quality has not been acquired.

To explore the influence of processing parameters on gear ultrasonic grinding, tangential ultrasonic vibration-assisted forming grinding gear was used to compare the grinding force, grinding temperature, surface roughness, and surface micromorphology in conventional grinding. The results of this study are proposed to provide a reference for the application of ultrasonic grinding in gear machining.

## 2. Experiment Setup and Methodology

### Experimental Setup and Conditions

**Figure 1:**
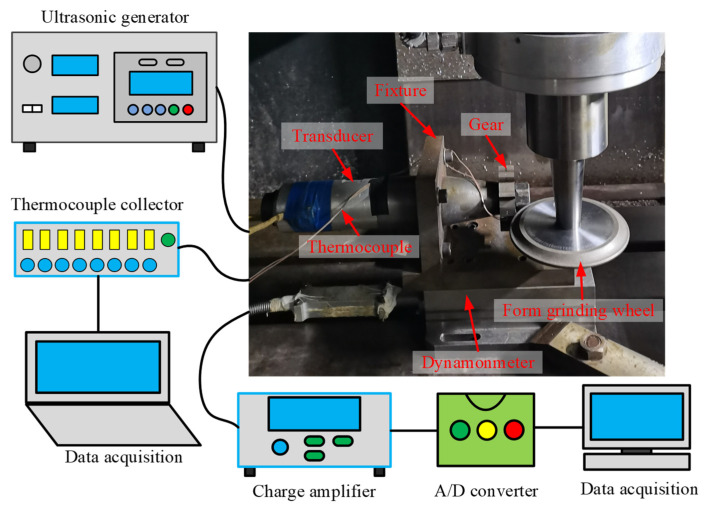
Experiment setup.

Conventional form grinding and tangential ultrasonic-vibration assisted forming grinding tests were carried out on a modified CNC machine center (VMC850E, SMTCL USA Inc., Shenyang Machine Tool Co. Ltd., Shengyang, China). The experimental setup is presented in Figure 1. It was mainly composed of a machine tool spindle, form-grinding wheel, ultrasonic vibration system, grinding force, and grinding temperature measurement system. During the tests, an electroplating CBN form-grinding wheel was connected to the machine tool spindle by the shank. The ultrasonic vibration system consisted of an ultrasonic generator, transducer, horn, and gear. The ultrasonic generator had a maximum output power of 500W and converted the alternating current into high-frequency electric oscillation for the ultrasonic vibration system. Next, the piezoelectric transducer converted the electric oscillation into mechanical vibrations at a high frequency. However, the amplitude of the mechanical vibration generated by the transducer was generally too low to be used for mechanical machining. Consequently, the horn was added to amplify the ultrasonic vibrational amplitude to applicable values. The gear material is 12Cr2Ni4A, with a module of 3 mm, 21 gear teeth, and a width of 20 mm. The ultrasonic vibration system was connected to the dynamometer (Kistler 9257B, Kistler Instrument Corp., Winterthur, Switzerland) via a fixture. The dynamometer was fixed to the workbench with a clamp. 

A dynamometer was used to measure the grinding force along the *x*, *y,* and *z* directions. An amplifier (5070A) was utilized to amplify the electrical signal from the dynamometer, which was fed to a data recorder (2825A). The recorded data were then saved and displayed on a computer using the Dynoware commercial software package (Kistler Instrument Corp).

The thermocouple method was utilized to measure the grinding temperature. Blind holes (with a diameter of 1 mm) were prefabricated in both tooth surfaces of the reference circle. The distance between the end of the blind hole and the upper tooth surface was 0.2 mm. Two K-type thermocouples were positioned inside the blind hole and touched its end, with plasticine sealing its opening to fix the thermocouple. A digital thermometer (HR-USB-T008, Shanghai Horizon Electronic Technology Co. Limited, Shanghai, China) was utilized to collect signals from the thermocouples. During the test, the maximum measured temperature was selected as the grinding temperature to reduce the heat accumulation effect on the measured temperature.

In the experiments, the TUVA-FGG and C-FGG were achieved by switching on or off the ultrasonic generator. However, in the ultrasonic vibration system, the range of the variation of resonant frequency was quite narrow once the dimensions of the machined gear were ascertained. Therefore, during the experiment, the ultrasonic frequency was regarded as constant. The experimental parameters are presented in Table 1. 

Each test was repeated three times, and a final value was obtained by averaging the measured results. After both tests, the machined gear teeth were cut using wire-electrode cutting to measure the surface roughness with a white-light interferometer (GT-K, BRUKER, Billerica, MA, USA). The residual surface stress was explored with the PROTO X-ray via the XRD method. The surface micromorphology was observed by an optical microscope (VHX-2000, KEYENCE, Osaka, Japan). The SEM micrographs were obtained through a scanning electron microscope (Merlin Compact, ZEISS, Oberkochen, Germany) to observe the surface microstructure of the gear. To gain more reliable data, three points were gauged for each set of parameters, and the average value was taken as the final result. 

## 3. Results and Discussion

### 3.1. Grinding Force

**Figure 2:**
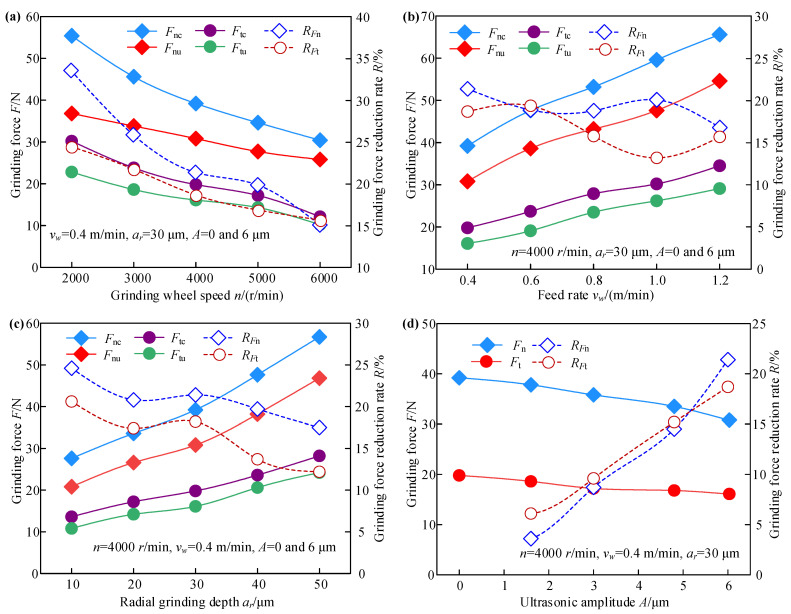
Effects of processing and ultrasonic parameters on the grinding force: (**a**) grinding wheel speed, (**b**) feed rate, (**c**) radial grinding depth, and (**d**) ultrasonic amplitude.

Figure 2 presents the effect of the processing and ultrasonic parameters on the grinding force in TUVA-FGG and C-FGG. To quantitatively describe the variation in grinding force, the coefficient *R_F_* was introduced to denote the reduction percentage in grinding force for TUVA-FGG compared to C-FGG under the same conditions. It can be expressed as follows:

(1) $$R_{F}=\frac{F_{c}-F_{u}}{F_{c}}$$

where *F_c_* and *F_u_* are grinding forces in C-FGG and TUVA-FGG, respectively. In Figure 2, *F*nc and *F*nu are the normal grinding force in C-FGG and TUVA-FGG, respectively; *F*tc and *F*tu are the tangential grinding force in C-FGG and TUVA-FGG, respectively; *R_F_*_n_ and *R_F_*_t_ denote the grinding force reduction of normal and tangential grinding force. 

As seen in Figure 2a, if the radial grinding depth and feed rate are kept constant, the normal and tangential grinding forces in both processes decrease with the grinding wheel speed. Compared with the C-FGG, the normal grinding force decreased by 33.6%, and the tangential grinding force dropped by 24.5% as the grinding wheel speed increased in the TUVA-FGG. This was attributed to the growing grinding wheel speed increasing the number of effective abrasive particles and decreasing the undeformed chip thickness. In addition, under the action of tangential ultrasonic vibration, intermittent cutting leads to the separation stage between the abrasive particle and the workpiece, which leads to a reduction in the grinding force. As presented in Figure 2b, the normal and tangential grinding forces in both cases appeared to increase with the feed rate, while under the action of ultrasonic vibration, the maximum reductions of normal and tangential grinding forces were 21.4 and 19.7%, respectively. Although the material removal rate increased with the feed rate, the number of dynamic abrasive particles resulted in an increase in the grinding force. Under the effect of ultrasonic vibration, the abrasive grinding arc length was enhanced, and corresponding to conventional grinding, it was equivalent to reducing the undeformed chip thickness, resulting in a decrease in the grinding force. According to Figure 2c, both normal and tangential grinding forces increase with radial grinding depth during both processes. Under the action of ultrasonic vibration, the maximum reductions of the two forces were about 24.6 and 20.3%, respectively. The main reason was that the effective dynamic abrasive particle number in the grinding zone increased with radial grinding depth and the corresponding grinding depth, which led to an increase in grinding contact arc length and grinding resistance. However, the abrasive particle would produce a larger instantaneous acceleration and impact the workpiece under ultrasonic vibration, weakening the grinding resistance. Therefore, both normal and tangential grinding forces exhibited a decreasing trend in tangential ultrasonic vibration-assisted forming grinding. As shown in Figure 2d, with increased ultrasonic amplitude, the maximum normal and tangential grinding forces decreased by 21.4 and 18.7%, respectively. With increased ultrasonic amplitude, the arc length of a single abrasive grain increased, and the undeformed chip thickness decreased. In addition, ultrasonic vibration can effectively reduce the friction coefficient between the abrasive particles and the workpiece, and the instantaneous impact has a softening effect on the material to a certain extent, leading to a reduction in the grinding force.

### 3.2. Grinding Temperature

**Figure 3:**
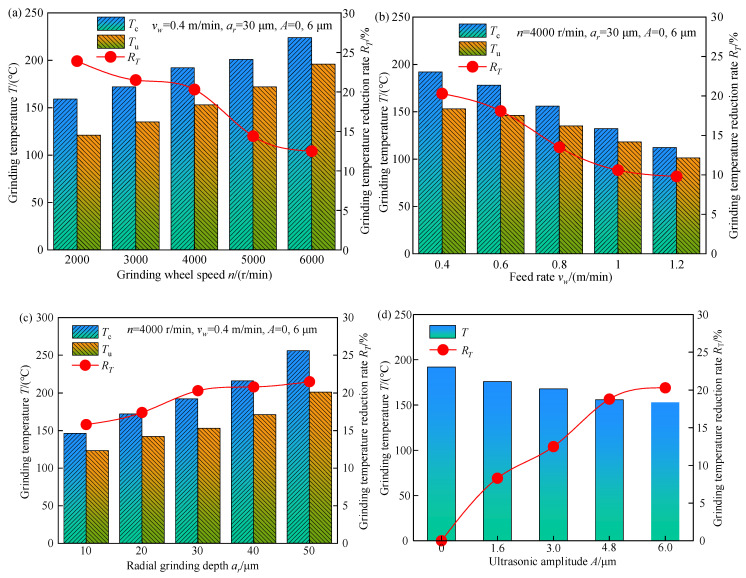
Effects of processing and ultrasonic parameters on the grinding temperature: (**a**) grinding, wheel speed, (**b**) feed rate, (**c**) radial grinding depth, and (**d**) ultrasonic amplitude.

The influence of processing parameters on grinding temperature during both processes is shown in Figure 3. 

In Figure 3, *T_c_* and *T_u_* represent the grinding temperatures of C-FGG and TUVA-FGG, respectively, and *R_T_* is the grinding temperature reduction rate in the latter compared with the former under the same conditions, and it can be calculated as

(2) $$R_{T}=\frac{T_{c}-T_{u}}{T_{c}}$$

Figure 3a shows that the grinding temperature increased gradually with the grinding wheel speed. The grinding temperature decreased by 12.1–23.9% during TUVA-FGG compared with C-FGG. This is mainly because with increasing grinding wheel speed, the number of heat pulses between the grinding grain and tooth surface increased, and the heat flux density in the grinding area increased. Under the action of ultrasonic vibration, there was a separation between abrasive particles and the workpiece, speeding up the heat loss to a certain extent and resulting in a large reduction in the grinding temperature. Figure 3b shows that the grinding temperature decreased with the feed rate. The main reason was that the increased feed rate raised the heat source speed moving on the tooth surface relative to the increase in heat flux in the grinding arc zone, leading to a reduction in grinding temperature. Compared with C-FGG, the grinding temperature can be reduced by 9.8–20.3% in TUVA-FGG. Figure 3c shows that the grinding temperature increased with the radial grinding depth. This can be attributed to the fact that the cutting thickness of a single grain increased with radial grinding depth, requiring more energy to remove the material and leading to a temperature rise. However, under the action of ultrasonic vibration, the contact between abrasive particles and the workpiece changed from continuous to intermittent contact, and the abrasive particle cutting angle increased, enhancing the cutting effect of abrasive particles and reducing the grinding temperature by 15.8–21.5%. As shown in Figure 3d, the grinding temperature dropped with the ultrasonic amplitude, and the maximum reduction was approximately 21.5%. This was mainly because with the increased ultrasonic amplitude, the softening effect of ultrasonic vibration was gradually enhanced, and the contact time between the abrasive grain and the workpiece was shortened; that is, the separation characteristics between them increased, which led to a reduction in grinding temperature.

### 3.3. Residual Stress

**Figure 4:**
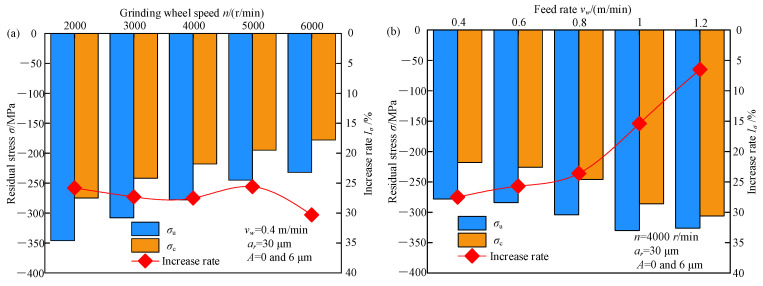

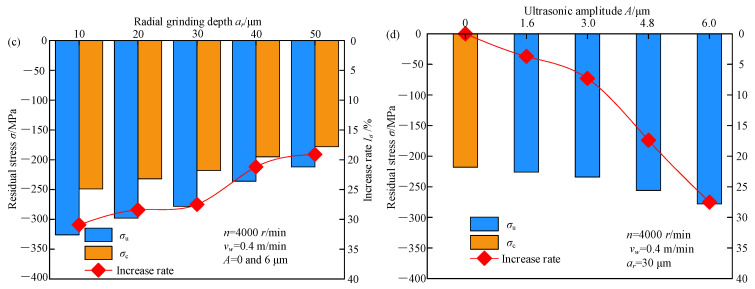
Effects of processing and ultrasonic parameters on the residual stress: (**a**) grinding wheel speed, (**b**) feed rate, (**c**) radial grinding depth, and (**d**) ultrasonic amplitude.

The effect of machining parameters on residual stress in both processes is shown in Figure 4. *σ*_u_ and *σ*_c_ denote the residual stress in TUVA-FGG and C-FGG, respectively. 

Figure 4a shows that the residual compressive stress of both processes decreased with the grinding wheel speed. With the increased grinding wheel speed, the reduction in grinding force led to a decrease in residual compressive stress caused by mechanical forces, while the increased grinding temperature led to an increase in residual tensile stress caused by the thermal effect. Therefore, under the effect of thermomechanical coupling, the residual compressive stress tended to decrease. Compared to C-FGG under the effect of ultrasonic vibration, the abrasive grain contact arc length could be prolonged, reducing the undeformed chip thickness and improving material plastic removal. Simultaneously, the interference of the adjacent grains led to grain reciprocating ironing the tooth surface. In addition, the vibration stress generated by the ultrasonic vibration applied to the gear could offset the internal stress of the workpiece to a certain extent, resulting in the reduction of the yield limit of the material so that the residual compressive stress on the tooth surface increased by approximately 25.8–30.3%. Figure 4b shows that in both cases, with the increased feed speed, the residual compressive stress during C-FGG exhibited a rising trend, while TUVA-FGG showed a trend of first increasing and then decreasing. Compared with the former, the residual compressive stress in the latter case increased by 6.5–27.5%. The main reason was that the separation between the abrasive grains and the workpiece weakened as the feed rate increased to a certain extent, resulting in the tendency of increasing residual compressive stress deterioration. Figure 4c shows that the residual compressive stress in both cases decreased with increasing radial grinding depth. However, when the radial grinding depth increased to a certain extent, the increasing trend of residual stress saturated. This was mainly because the grinding force and grinding temperature increased with the radial grinding depth. At a smaller radial grinding depth, the grinding force played a dominant role. At larger grinding depths, the residual tensile stress induced by the thermal effect increased, as shown in Figure 4. The residual stress increased greatly when the grinding depth was small. Under the action of ultrasonic vibration, abrasive grains could strengthen the tooth surface to a certain extent and weaken the thermal effect so that the overall residual stress increased by 19.1–30.9%. Figure 4d illustrates the ultrasonic amplitude effect on residual stress in both processes. With the increased ultrasonic amplitude, the residual compressive stress grew by 3.7–27.5%. Under the action of ultrasonic vibration, the reciprocating ironing effect of abrasive grains on the surface resulted in an increasing trend of residual stress.

### 3.4. Surface Roughness

**Figure 5:**
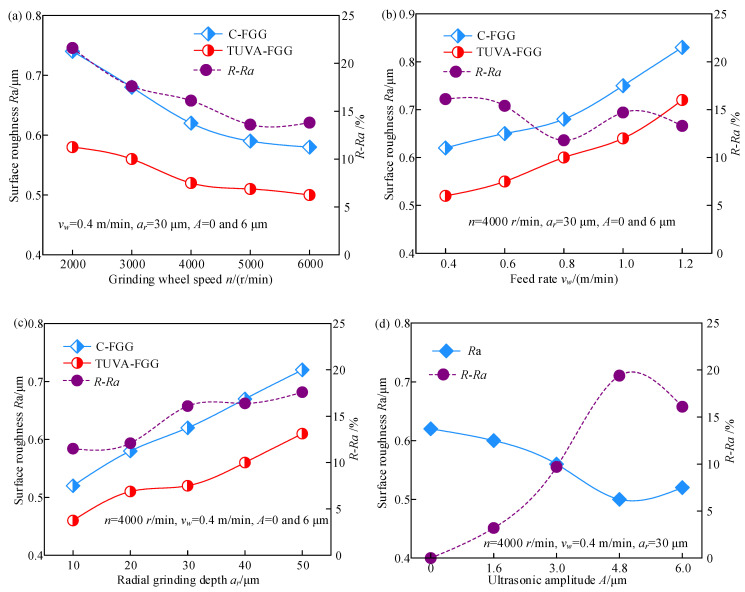
Effects of processing and ultrasonic parameters on the surface roughness: (**a**) grinding wheel speed, (**b**) feed rate, (**c**) radial grinding depth, and (**d**) ultrasonic amplitude.

The effect of processing and ultrasonic parameters on surface roughness is depicted in Figure 5, where *R − Ra* denotes the surface roughness reduction ratio of the TUVA-FGG to C-FGG, and it can be calculated as

(3) $$R-Ra=\frac{R_{c}-R_{u}}{R_{c}}$$

where *R_c_* and *R_u_* are the surface roughness in C-FGG and TUVA-FGG, respectively.

As shown in Figure 5a, the surface roughness decreases with increasing grinding wheel speed. Compared with C-FGG, the surface roughness decreased by approximately 13.6–21.6% under the action of ultrasonic vibration. The main reason is that the dynamic abrasive grains involved in grinding per unit of time increased with grinding wheel speed, leading to a decreased grinding depth of a single abrasive grain and a decrease in surface roughness. As shown in Figure 5b, with the increased feed rate, the surface roughness of both cases exhibited an increasing trend. The surface roughness during TUVA-FGG decreased by approximately 11.8–16.1% compared with C-FGG. This was mainly because the contact time shortened with increased feed rate, reducing the number of dynamic grinding grains and increasing the amount of residual material after grinding. However, during TUVA-FGG, the grain movement length increased, and the interference with adjacent grains was strengthened, promoting the material removal and, thus, reducing the surface roughness. As shown in Figure 5c, with the increased radial grinding depth, the surface roughness showed an increasing trend. Compared to C-FGG, the surface roughness decreased by 11.5–17.6% under ultrasonic vibration. This was mainly because the undeformed chip thickness of a single grain increased with the radial grinding depth, producing plastic bulges of material on either side of the grain while under the action of ultrasonic vibration, and the undeformed chip thickness was enhanced and improved surface roughness at larger radial grinding depths. Figure 5d shows that the surface roughness first decreased and then increased with increased ultrasonic amplitude. Compared with C-FGG, the surface roughness decreased by 3.2–19.4%. This was mainly attributed to the fact that the separation of the abrasive grain workpiece was enhanced with increased ultrasonic amplitude, effectively improving the material removal and the grain reciprocating ironing effect, resulting in lower surface roughness. However, with a further increase in the ultrasonic amplitude, the grain impact was enhanced and coupled with the softening effect on the material. Thus, the material plastic removal was improved, resulting in a slightly increased surface roughness.

### 3.5. Micromorphology of the Tooth Surface 

**Figure 6:**
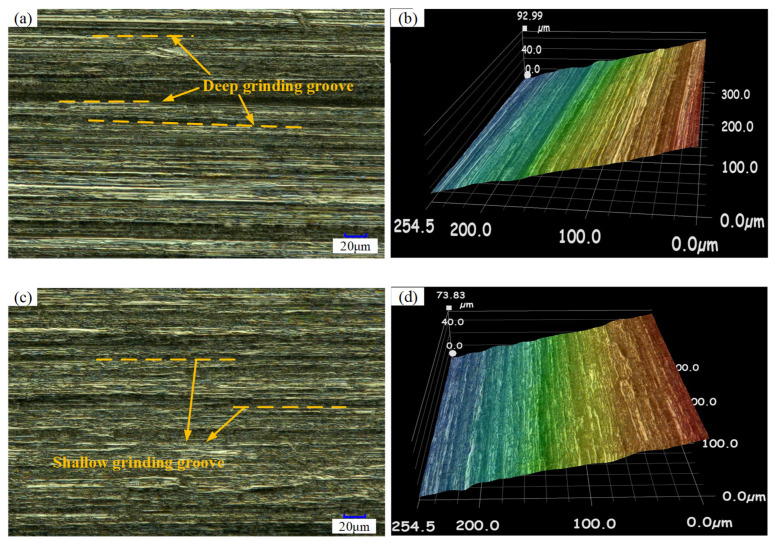
Surface morphology: (**a**,**b**) C-FGG, (**c**,**d**) TUVA-FGG.

Figure 6 shows the tooth surface topography in the C-FGG and TUVA-FGG processes. During the C-FGG processing, as shown in Figure 6a,b, deep grinding grooves and ridges clearly appeared on the gear surface parallel to the grinding direction. The grinding groove was shallow under the action of ultrasonic vibration, and the abrasive grain paths overlapped. In TUVA-FGG, the relationship between the abrasive grains and the workpiece was changed with the grains’ reciprocating impact on the workpiece. Therefore, the surface during TUVA-FGG presented a smoother appearance. 

**Figure 7:**
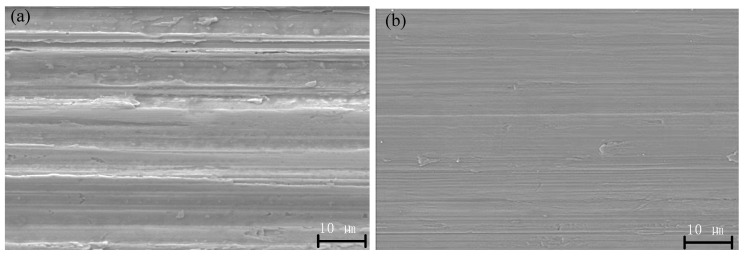
Typical SEM micrographs of the gear tooth surface: (**a**) C-FGG and (**b**) TUVA-FGG.

SEM analysis was also performed on TUVA-FGG finished gear workpieces to better investigate the morphology and mechanism of the TUVA-FGG process. Figure 7a clearly indicates that during the C-FGG process, the gear tooth surface contained deep grinding grooves, chipping, and severe deposition. The micrograph with TUVA-FGG surface morphology is shown in Figure 7b. The TUVA-FGG machined surface presented a smooth appearance, while shallow grinding grooves, slight deposition, and ironing effects could be identified. In view of the uneven distribution of abrasive grains, the material would not be completely fractured when quickly cut, leaving material residue on the surface. During the TUVA-FGG process, the separation characteristics of abrasive grains and the workpiece led to intermittent cutting, reducing the grinding force while the grains’ cut-in angle was changed, and its transient impact on the material was improved. This caused the instantaneous concentration of energy in the grain and material contact area and induced a stress wave enhancing the material removal effect. Meanwhile, under ultrasonic vibration, the succeeding abrasive grains ironed the surface, improving the surface quality. 

## 4. Conclusions

In this study, tangential ultrasonic vibration-assisted forming grinding gear tests were conducted by applying ultrasonic vibration to the gear workpiece. The processing effects, including grinding force, grinding temperature, residual stress, surface roughness, and surface morphology, were compared to those of the conventional forming grinding gear. Based on the findings, the following conclusions can be drawn:

(1) The tangential ultrasonic vibration was beneficial for reducing the grinding force, grinding temperature, and surface roughness. With the increase in grinding wheel speed, the normal grinding force and tangential grinding force, grinding temperature, and surface roughness maximum decreased by 33.6, 24.5, 23.9, and 21.6%, respectively. With the increase in feed rate, the maximum reduction was 21.4, 19.7, 20.3, and 16.1%, respectively. With the increase in radial grinding depth, the corresponding maximum reductions were 24.6, 20.3, 21.5, and 17.6%, respectively. With the increase in ultrasonic amplitude, the maximum normal force and tangential force decreased by 21.4 and 19.7%, respectively, the maximum grinding temperature decreased by 21.5%, and the maximum surface roughness decreased by 19.4%.

(2) The TUVA-FGG effectively increased the residual compressive stresses and improved the micromorphology of the tooth surface compared with conventional grinding. The maximal residual compressive stresses grew by 30.3, 27.5, 30.9, and 27.5% with the variation in grinding wheel speed, feed rate, radial grinding depth, and ultrasonic amplitude, respectively. The micromorphology of the tooth surface became smoother and more uniform under the action of ultrasonic vibration.

## Figures and Tables

**Table 1:** Experimental parameters.

Type	Parameters	Value
Grinding wheel parameters	Diameter of the grinding wheel *D* (mm)	110
	Grinding wheel granularity (#)	400
Grinding parameters	Grinding wheel speed *n* (r/min)	2000, 3000, 4000, 5000, 6000
	Grinding depth *a_r_* (μm)	10, 20, 30, 40, 50
	Workpiece feed rate *v_w_* (m/min)	0.4, 0.6, 0.8, 1.0, 1.2
	Coolant	None
Ultrasonic parameters	Ultrasonic amplitude *A* (μm)	0, 1.6, 3.0, 4.8, 6.0
	Ultrasonic frequency *f* (kHz)	20.17

## Data Availability

Not applicable.
